# The vexing relationship between socioeconomic status and health

**DOI:** 10.1186/s13584-020-00430-0

**Published:** 2020-11-26

**Authors:** J. Travis Donahoe, Thomas G. McGuire

**Affiliations:** 1 Health Policy, Harvard University, Boston, USA; 2 Health Economics, Harvard Medical School, Boston, USA

**Keywords:** Health disparities, Socioeconomic status and health, Causality

## Abstract

In a recent issue of this *Journal*, Politzer, Shmueli, and Avni estimate the economic costs of health disparities due to socioeconomic status (SES) in Israel (Politzer et al., Isr J Health Policy Res 8: 46, 2019). Using three measures of SES, the socioeconomic ranking of localities, individual income, and individual education, Politzer and colleagues estimate welfare loss due to higher mortality, productivity loss due to poorer health, excess health care treatment costs, and excess disability payments for individuals with below median SES relative to those with above median SES. They find the economic costs of health disparities are substantial, adding up to between 1.1 and 3.1 billion USD annually—between 0.7 and 1.6% of Israel’s GDP.

This paper is useful and informative. It is, to our knowledge, the first comprehensive quantification of the economic costs stemming from health disparities in Israel. In spite of many social policies designed to level economic opportunity and social welfare generally, by most measures, Israel is among the most unequal in the distribution of income among all OECD countries (Cornfeld and Danieli, Isr Econ Rev 12:51–95, 2015). Politzer and colleagues expose the magnitude and sources of health-related loss that Israel faces because of such inequality and shows how the costs of inequality are borne to some degree by all members of society. This short commentary discusses the complicated relationship between SES and health and puts the findings from Politzer and colleagues in the context of the international literature on the subject.

## Main text

The inverse relationship between SES and health is one of the most important and complicated issues in social policy. Poorer SES, whether measured by education, income, occupation, race and ethnicity, or locality, has been associated a variety of negative health outcomes including shorter life expectancy [1], worse mental health [2], higher mortality from a wide-range of diseases [3], worse health behaviors [4], and most recently, higher mortality from COVID-19 [5]. Two dominant models seek to explain the robust negative relationship between SES and health.

First, SES can be one factor influencing health. There are multiple pathways through which SES influences health, including its impacts on individual health behaviors and lifestyles, exposures to environmental stressors and toxins, and access to health care [6]. Going a step further, the SES-to-health model argues that SES is a fundamental cause of health because it embodies all of the resources one has available to avoid risks and minimize the effects of diseases [7]. The second dominant model sees the causal arrow as running in the other direction. The social selection model argues that poor health, due to genetics or one’s environment, negatively affects SES and is responsible for the inverse association.

Thirty years ago, in a classic paper in epidemiology, Dohrenwend and colleagues, using a birth cohort sample of 4914 Israel-born adults of European and North African ethnicities, tested whether the relationship between SES and psychiatric disorders was due to SES causing health or social selection [8]. Dohrenwend and colleagues’ insight was that ethnicity, an element of SES, cannot be an effect of a psychiatric disorder. Thus, conditional on non-ethnic measures of SES (education and occupation in the paper), higher prevalence of psychiatric disorders among Israelis of North African descent relative to Israelis of European descent supports the SES-to-health model and, in particular, the marginalization of North Africans in Israel causing higher rates of psychiatric disease.

By contrast, higher prevalence of psychiatric disorders among Israelis of European descent with the same level of SES supports the selection model. Because Israelis of European descent face less prejudice and discrimination in Israel relative to Israelis of North African descent, the social selection model predicts that they will be able to achieve higher SES than Israelis of North African descent with the same health and psychiatric illness status. Thus, under the social selection model, Israelis of European descent will have higher prevalence of psychiatric disease than Israelis of North African descent with the similar occupations and levels of education.

Using the methodology outlined above, Dohrenwend and colleagues found that the inverse relationship between SES and schizophrenia was explained by the social selection model while the inverse relationship between SES and depression, antisocial personality, and substance use disorders was accounted for by the causal SES-to-health model. Dohrenwend and colleagues demonstrated that the relationship between SES and health is a two-way street, depending on the illness and depending on the dimension of SES being considered.

Today, there is a flourishing international literature which applies rigorous experimental and quasi-experimental methods to isolate the causal impacts of SES on health and the debate is still as vigorous as ever. Consider just the causal effects of education on mortality. The relationship between education and mortality has been the subject of numerous quasi-experimental studies that exploit changes in compulsory schooling laws in the US [9–11], Sweden [12, 13], the UK [14, 15], the Netherlands [16], and France [17]. Some studies find higher education substantially lowers mortality rates [9–13, 15, 16, 18], yet others find education has no effect [14, 17, 19]. In a recent review of the education and mortality literature, Galama, Lleras-Muney, and van Kippersluis conclude that there is not a single causal effect of education on mortality but that there is strong evidence for an effect of education on mortality in certain times and in certain places [20]. Similar debates in the literature rage over the extent to which income [21–23] and income inequality are causally related to health [24, 25].

It is this vexing area of social policy in which Politzer, Shmueli, and Avni’s paper presents estimates on the economic burden of health disparities by SES [26]. The innovation and usefulness of Politzer, Shmueli, and Avni’s paper is that it presents a thorough analysis of the social costs stemming from health disparities, treating SES as a fundamental cause of health. However, as the authors recognize, attributing the entire association between SES and health costs to an SES causal connection might overestimate the true relationship between health disparities by SES and economic costs. Accounting for this, Politzer, Shmueli, and Avni also made conservative assumptions that offset this and might underestimate the costs of health disparities. In particular, the authors only consider the health costs for below median SES individuals relative to above median SES individuals and do not incorporate the costs of health disparities that accrue continuously along the socioeconomic gradient.

Some of the costs which Politzer, Shmueli, and Avni estimate reflect mostly the causal portion of the SES-to-health relationship. The estimates of welfare loss from excess mortality for Israelis in below vs. above median SES localities are likely explained in large part by causal effects of lower SES areas on health, through the effects of lower SES areas in generating worse health behaviors, more exposure to environmental stressors and toxins [27], and worse performing health systems [28]. On the other hand, and acknowledging that this is a hotly contested point, worse health for individuals with below median vs. above median income likely reflects mostly the reverse-causal part of the SES-and-health relationship [22].

Understanding the precise nature of the social cost of health disparities would require integrating Politzer, Shmueli, and Avni’s methodology with estimates of the true causal effects of SES on health. However, as this commentary describes, such causal effects vary according to the nature of the disease being considered and the precise context in which the causal effect was estimated. Politzer, Shmueli, and Avni’s work takes a step back from this puzzle and shows us that under a reasonable set of assumptions, the costs of health disparities in Israel are large in magnitude. After-tax income inequality is high in Israel realtive to other OECD countries [29]. Policymakers should consider the economic costs of health disparities when evaluating the merits of policies that would lessen or exacerbate existing inequalities. 
